# The role of preoperative D-dimer levels in diagnosing adnexal torsion in children and adolescents: a prospective monocentric pilot analysis

**DOI:** 10.3389/fmed.2025.1706633

**Published:** 2025-11-20

**Authors:** Alessandro Boscarelli, Manuela Giangreco, Elena Valvo, Daniela Codrich, Virginia Masiello, Marianna Iaquinto, Maria-Grazia Scarpa, Damiana Olenik, Sonia Maita, Carmen Campilongo, Edoardo Guida, Jürgen Schleef

**Affiliations:** 1 Department of Pediatric Surgery and Urology, Institute for Maternal and Child Health - IRCCS “Burlo Garofolo, ” Trieste, Italy; 2 Clinical Epidemiology and Public Health Research Unit, Institute for Maternal and Child Health - IRCCS “Burlo Garofolo, ” Trieste, Italy; 3 University of Trieste, Faculty of Medicine and Surgery, Trieste, Italy

**Keywords:** adnexal torsion, adolescents, children, D-dimer levels, diagnosis

## Abstract

**Introduction:**

The diagnosis of adnexal torsion in children remains nonspecific. In some studies, D-dimer was shown to be a promising biochemical marker, especially in animals. This analysis aimed to preliminarily evaluate the specificity and sensitivity of D-dimer levels in predicting the diagnosis of adnexal torsion at our institution.

**Methods:**

All female patients who presented at the emergency department from January 2022 to December 2024 with symptoms suggestive of adnexal torsion were prospectively enrolled in the study. Preoperative D-dimer levels were obtained for all patients undergoing surgical exploration. Descriptive analysis was computed, and a receiver operating characteristic curve was constructed via univariate logistic regression.

**Results:**

Of the 27 eligible patients, 15 (aged 7–17 years) participated in the study. Torsion was found on the left side in six patients (40%) and on the right side in nine patients (60%). Almost all patients (80%) were treated laparoscopically. No postoperative complications were encountered. None of the examined variables were significantly correlated with adnexal torsion. Notably, D-dimer levels were higher in patients with adnexal torsion (odds ratio = 1, *p* = 0.29), with a sensitivity of 0.86 and a specificity of 0.75.

**Conclusion:**

No association was observed between D-dimer levels and ovarian torsion in our cohort. Nonetheless, the sensitivity and specificity results lead us to believe that preoperative D-dimer levels could help surgeons in more accurately predicting the possibility of an adnexal torsion in children and adolescents, but this should not replace imaging studies and diagnostic laparoscopy especially in doubtful cases.

## Introduction

Adnexal torsion, also known as ovarian torsion, is the fifth most common gynecologic emergency, with approximately 30% of cases occurring in females aged under 20 years. Its incidence is estimated at 5 per 100,000 females aged 1–20 years, with girls aged over 10 years at increased risk due to hormonal influences and gonadal growth leading to a higher frequency of both physiological and pathological masses. The right ovary is most frequently involved, and a delayed identification of adnexal torsion may lead to hemorrhagic infarction and necrosis of adnexal structures, which may impair future fertility (1–3).

The clinical presentation of adnexal torsion is often nonspecific, with the most common symptom being acute, intermittent, non-radiating lower abdominal pain, associated with nausea and vomiting in 60%−70% of patients. Clinical signs such as rebound tenderness and peritoneal irritation are less commonly observed. Laboratory markers, including leukocytosis, pyuria, C-reactive protein (CRP), and erythrocyte sedimentation rate, are by no means reliable for diagnosing adnexal torsion.

Diagnostic imaging primarily involves abdominal ultrasonography (US), which demonstrates high sensitivity (92%) and specificity (96%) in detecting adnexal torsion. In cases where US is inconclusive, magnetic resonance imaging (MRI) and/or computed tomography (CT) may be utilized, although they are less commonly employed. However, no clinical or imaging criteria are sufficiently definitive to confirm the preoperative diagnosis of adnexal torsion. Therefore, patients with a high clinical suspicion still undergo emergent diagnostic laparoscopy (1, 3).

In adults, the inflammatory marker interleukin 6 (IL6) has shown promise in predicting adnexal torsion. A few studies have also investigated the role of other biochemical markers, such as D-dimer, in diagnosing adnexal torsion. D-dimer, a fibrin degradation product, has been proposed as a potential indicator of ischemia in various tissues, including the ovaries. Preliminary data suggest that elevated D-dimer levels may correlate with adnexal torsion, although further studies are needed to establish its diagnostic utility in children and adolescents (4, 5).

This study aimed to prospectively investigate the specificity and sensitivity of preoperative D-dimer levels in predicting the diagnosis of adnexal torsion in children and adolescents, potentially reducing unnecessary surgical procedures and preserving ovarian function in affected patients.

## Methods

A multicenter prospective observational study of diagnostic appropriateness was approved by the institutional review board of our hospital (approval number: CEUR-2021-Os-219), and informed consent was obtained from all study participants. In this preliminary monocentric analysis, we enrolled female patients aged under 18 years who presented to the emergency department with symptoms and imaging findings suggestive of adnexal torsion between January 2022 and December 2024. The exclusion criteria were age 18 years and older, a history of prior surgery for adnexal pathologies, and exhibiting clinical symptoms and imaging findings suggestive of alternative surgical conditions, such as appendicitis or gastroenteritis.

The primary aim was to evaluate the diagnostic accuracy of serum D-dimer levels in the preoperative diagnosis of adnexal torsion. The secondary aim was to investigate the possible reduction of unnecessary surgery and any related complications in these patients.

A Word-based data sheet and an Excel spreadsheet were used to collect the patients' data, including age; clinical history; preoperative examination values, symptoms, and imaging features; laparoscopic findings (e.g., the aspect of each tube and the ovaries), presence of cyst or malformations; surgical outcomes; and complications. Plasma D-dimer was measured at admission using a latex-based automated immunologic kit HemosIL D-dimer HS 500 (Cat. 0020500100, Werfen Instrumentation Laboratory Company, United States) on the system ACLTOP350^CTS^ with a sensitivity of 100% (95.2% - 100%, 95% CI) and a specificity of 42.3% (35.7% - 49.1%, 95% CI). The clinical cut-off value was settled at 500 ng/mL of D-dimer.

The patients' demographic and clinical characteristics were analyzed descriptively, with categorical variables reported as the number (percentage) of the subjects, and quantitative variables reported as the median (interquartile range). The association between categorical variables was evaluated by the Chi-square test or the exact Fisher test, as appropriate. The non-parametric Wilcoxon Mann-Whitney test was applied to assess a difference in the distribution of a continuous variable between the categories of a dichotomic variable. A receiver operating characteristic (ROC) curve was constructed to assess the accuracy of D-dimer levels in the preoperative diagnosis of adnexal torsion, which was used to calculate their sensitivity, specificity, and positive and negative predictive values. The optimal threshold of D-dimer levels capable of discriminating the presence of adnexal torsion was evaluated using the Youden Index. The number of operations that could have been avoided if D-dimer levels were used as a predictor of adnexal torsion was evaluated by calculating and verifying the number of patients who had a D-dimer level below the optimal threshold found using the Youden index. All analyses were conducted using the SAS 9.4 software (SAS Institute Inc., Cary, NC, USA).

## Results

Of the 27 eligible patients with suspected adnexal torsion, 12 were excluded from this study due to refusal to participate (*n* = 4, 33.3%), incorrect diagnosis (*n* = 4, 33.3%; three had appendicitis, and one had an isolated paratubal cyst), not undergoing surgical exploration (*n* = 3, 25.0%), and a prior surgery on the contralateral side (*n* = 1, 8.3%). Therefore, 15 patients were ultimately included in this preliminary monocentric analysis, and their clinical characteristics are summarized in Table 1. Their median age was 13 years (IQR: 11-15; range: 7–17). All patients reported abdominal pain, as well as other symptoms, including vomiting (*n* = 7, 46.7%), nausea (*n* = 4, 26.7%), fever (*n* = 3, 20.0%), and abdominal mass (*n* = 3, 20.0%). Interestingly, at presentation, 12 patients (80.0%) reported menstruation.

**Table 1:** Clinical Characteristics of patients enrolled in our study (*n* = *number; N* = *no; Y* = *yes*).

	***n***	**%**
**Menstruation**		
N	3	20.00
Y	12	80.00
**Symptoms**		
Fever, Pain, Abdominal mass	2	13.33
Fever, Pain, Nausea	1	6.67
Pain	3	20.00
Pain, Nausea	2	13.33
Pain, Nausea, Vomiting	1	6.67
Pain, vomiting	5	33.33
Pain, vomiting, Abdominal mass	1	6.67
**Ultrasonography**		
N	0	0.00
Y	15	100.00
**Computed tomography**		
N	13	86.67
Y	2	13.33
**Magnetic resonance imaging**		
N	15	100.00
Y	0	0.00
**Suspected side**		
Left	6	40.00
Right	9	60.00
**Approach**		
Lap	12	80.00
Lap converted	1	6.67
Open	2	13.33
**Side**		
Left	7	46.67
Right	8	53.33
**Torsion**		
N	8	53.33
Y	7	46.67
**Torsion in prepubescents**	2	28.57
**Torsion in pubescents**	5	71.43
**Surgery**		
Cyst ablation	2	13.33
Derotation	1	6.67
Derotation + oophoropexy	2	13.33
Derotation + punction	1	6.67
Hemorrhagic corpus luteum cyst	2	13.33
Oophorectomy	2	13.33
Punction	1	6.67
Punction + cyst ablation	2	13.33
Punction + cyst ablation + derotation	1	6.67
Punction + oophorectomy	1	6.67
**Post-operative_complications**		
N	15	100.00
Y	0	0.00

According to the preoperative examinations, all patients underwent an abdominal US, and only two (13.3%) required a CT for a more accurate diagnosis. The suspected side was more frequently the right side (*n* = 9, 60%). Every patient with suspected adnexal torsion underwent surgical exploration: 12 (80.0%) underwent a laparoscopic surgery, 1 (6.7%) began laparoscopically but was then converted to open surgery, and 2 (13.3%) underwent open surgery. Intraoperatively, it was determined that the involved side was the left side in seven patients (46.7%) and the right side in eight patients (53.3%). Ultimately, only seven patients (46.7%) presented with an actual adnexal torsion, of whom two (28.57%) were in prepubertal age at presentation, while five (71.43%) were pubescent. Notably, in both prepubescent patients presenting with an adnexal torsion a specific reason for torsion was not found at intervention.

All patients underwent different surgical procedures according to the primary problem: one (6.7%) underwent a simple derotation of the adnexa, two (13.3%) underwent derotation of the adnexa and oophoropexy, one (6.7%) underwent derotation of the adnexa and punction of the cyst, two (13.3%) underwent oophorectomy, one (6.7%) only underwent punction of the cyst, two (13.3%) underwent ablation of the cyst, two (13.3%) underwent punction and ablation of the cyst, one (6.7%) underwent punction and ablation of the cyst and derotation of the adnexa, and one (6.7%) underwent punction of the cyst and oophorectomy; two (13.3%) presented only with a haemorrhagic corpus luteum cyst requiring no procedures. Given the importance of ovarian presenvation, no histological examinations were performed to define the damage caused by torsion in intraoperatively confirmed cases. Remarkably, none of these patients experienced postoperative complications. All patients underwent a US scan at 2 and 6 months after surgery.

**Figure 1:**
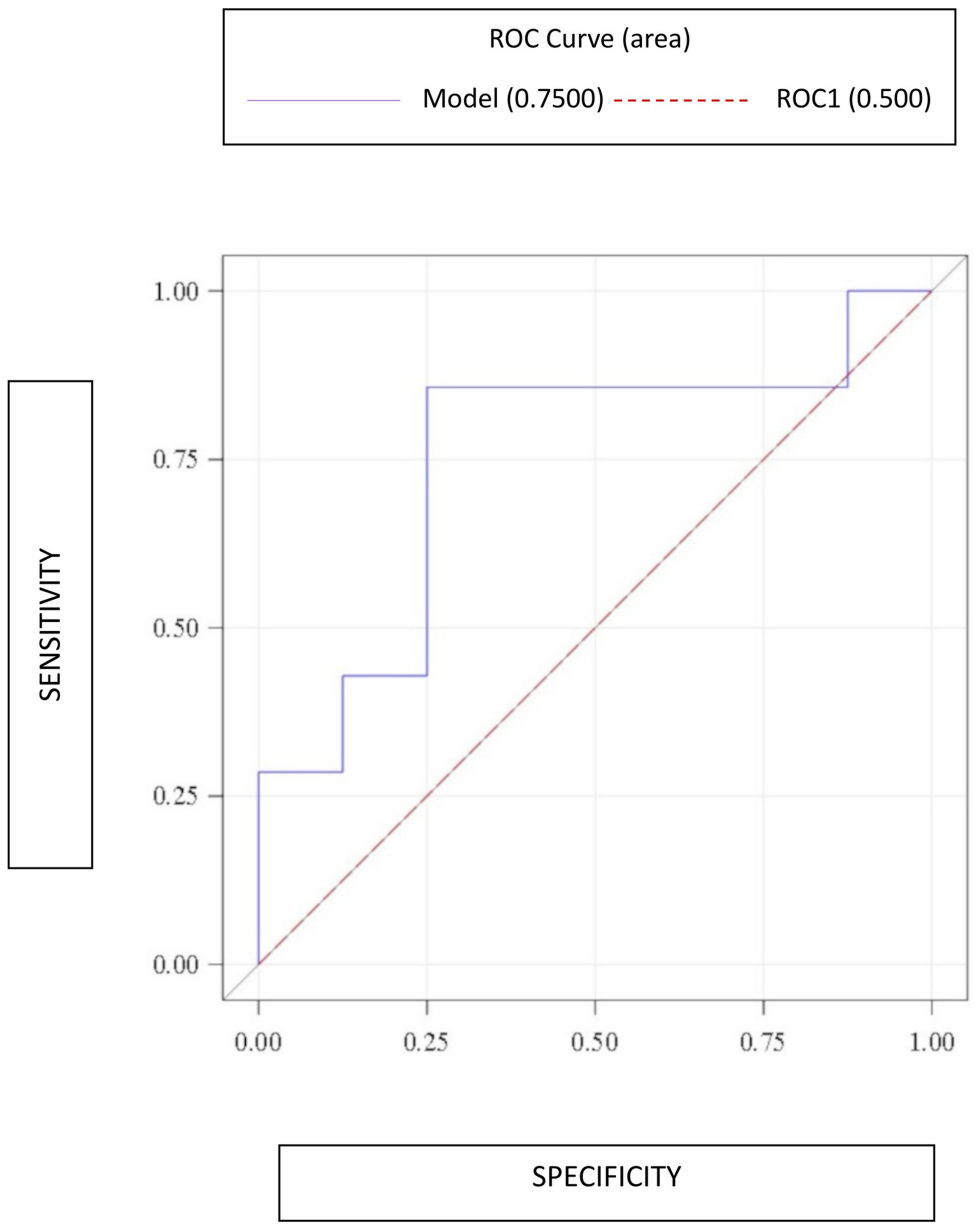
Receiver operating characteristic (ROC) curve constructed to evaluate the accuracy of D-dimer levels in the preoperative diagnosis of adnexal torsion.

Statistically, patients with and without adnexal torsion exhibited no important differences in the preoperative examinations (Table 2). Specifically, they had similar white blood cell counts [median: 8.9 × 10^3^/μL (IQR: 8.2-14.1 × 10^3^/μL) vs. 9.6 × 10^3^/μL (IQR: 8-10.6 × 10^3^/μL), *p* = 0.61], neutrophil percentages [median: 77.3% (IQR: 53.2-83.1%) vs. 72.3% (IQR: 63.7-79.2), *p* = 0.73], and CRP levels [median: 1.6 mg/L (IQR: 0.5-66 mg/L) vs. 1.7 mg/L (0.3-32.6 mg/L), *p* = 0.78]. However, preoperative D-dimer levels were higher among the patients with than without adnexal torsion [median: 753 ng/mL (IQR: 520-1887 ng/mL) vs. 385 ng/mL (IQR: 321.5-660 ng/mL), *p* = 0.14]. Notably, the association between preoperative D-dimer levels and adnexal torsion had an odds ratio (OR) of 1 (*p* = 0.29). The ROC curve had an area under the curve (AUC) = 0.75 (95% CI: 0.47-1.00), showing a sensitivity of 0.86 and specificity of 0.75. The optimal threshold of D-dimer levels was 520 ng/mL (Table 3 and Figure 1).

**Table 2:** Statistical analysis of demographic and preoperative blood values and their correlation with adnexal torsion.

	**Torsion**	***N***	**Min**	**25°percentile**	**Median**	**75°percentile**	**Max**	**Wilcoxon Mann Whitney *P*-value**
Age	N	8	10	11.5	12.5	15	17	0.91
	Y	7	7	7	14	16	16	
Weight	N	8	50	53.5	56.5	63	70	0.65
	Y	7	25	35	54	69	77	
WBC	N	8	5.9	8	9.6	10.6	13.4	0.61
	Y	7	6.5	8.2	8.9	14.1	15.9	
Neu	N	8	46.4	63.7	72.3	79.2	84.1	0.73
	Y	7	46.4	53.2	77.3	83.1	92.1	
CRP	N	8	0.2	0.3	1.7	32.6	140.3	0.78
	Y	7	0.2	0.5	1.6	66	98.7	
D-dimer	N	8	268	321.5	385	660	1,788	0.14
	Y	7	302	520	753	1,887	9,450	

n = number; N = no; Y = yes; WBC = White Blood Cells; Neu = Neutrophils; CRP = C-Reactive Protein.

**Table 3:** Statistical analysis to assess the accuracy of D-dimer levels in the preoperative diagnosis of adnexal torsion.

**D-dimer**	**Univariate model - Torsion**			
	**Odds ratio**	***p*** **-value**	**Sensitivity**	**Specificity**
	1.00	0.29	0,86	0,75

## Discussion

Adnexal torsion is defined as the partial or complete rotation of the adnexa on its vascular pedicle. It can involve the ovary, the fallopian tube, or both. The venous blood flow is the first to be impaired, followed by the arterial blood flow, leading to congestion, adnexal edema, ischemia, and, ultimately, necrosis (2).

According to the literature, diagnosing adnexal torsion is challenging due to the lack of distinctive symptoms, specific tests, or suggestive radiological findings. The clinical signs typically reported by patients experiencing an adnexal torsion include lower abdominal pain (~60%−70%), abdominal tenderness (~80%−90%), and fever (~10%, usually as a late finding resulting from the presence of necrotic tissue). A palpable mass is reported in 20%−36% of children presenting with adnexal torsion, and the cause of torsion of the ovarian masses is likely related to the increased size and weight of the involved ovary (2, 3, 6). Regarding laboratory tests, leukocytosis, pyuria, CRP level, and erythrocyte sedimentation are not helpful in diagnosing adnexal torsion (1). In contrast, according to the literature, D-dimer and IL6 levels could be informative in diagnosing adnexal torsion (4, 5).

Additionally, US findings can be indicative of adnexal torsion, including unilateral ovarian enlargement, ovarian edema characterized by the presence of a hyperechogenic stroma, free fluid, and coiled vascular pedicle (called the “whirlpool sign”) (1). Some pediatric patients may present with a normal blood flow in the Doppler scan if the ovary has transiently and spontaneously detorsed, is only partially torsed, or if the US study is performed early in the torsion process when arterial perfusion is still preserved and only the venous and lymphatic drainage are obstructed. Similarly, MRI may show a decreased contrast enhancement of the ovary, asymmetric ovarian enlargement, uterine deviation toward the pathologic side, and the presence of multiple small peripherally located follicles; however, MRI may require sedation, and it is not customarily available in an emergency care setting. Lastly, intra-adnexal CT features of torsion included uterine tube thickening (74%), eccentric or concentric wall thickening (54%), and eccentric septal thickening (50%) (1, 2); however, CT involves the use of X-rays and, thus, should only be used in selected pediatric cases.

Currently, in cases of suspected adnexal torsion, the patient must undergo an urgent laparoscopic exploration. Indeed, in our cohort, all enrolled patients underwent surgery, but only seven (46.7%) presented with an actual adnexal torsion requiring surgical correction. Considering that the goals of surgery are to detorse the adnexa and preserve the ovary regardless of its appearance and the timing of presentation (1), no histological examinations were performed in case of adnexal torsion. All patients were followed up with a US scan at 2 and 6 months, and a gynecological evaluation was suggested to all patients close to adolescence. Stimulatingly, indocyanine green (ICG) fluorescence imaging has recently revolutionized surgical practice across various specialties. Although ICG fluorescence is used more commonly in the adult population, its adoption in pediatric surgery continues to expand. In particular, in cases of adnexal torsion ICG started to be used to assess ovarian perfusion after detorsion, thus reducing the rate of oophorectomy (7). At our institution, ICG is currently being used to study the perfusion after detorsion in males experiencing testicular torsion, and it could be used to evaluate ovarian perfusion in torsed adnexa in the future as well.

Even if the inflammatory marker IL6 has shown promise in predicting adnexal torsion in adults (4), we decided to investigate exclusively the potential of D-dimer as a diagnostic biomarker due to the fact that unlike IL6, the D-dimer could be easily added to routine blood exams without any additional costs for the institution. Based on our current experience, patients presenting with an adnexal torsion were prone to having a higher D-dimer level than those without an adnexal torsion. Actually, our statistical analyses do not support the hypothesis that high D-dimer levels are suggestive of adnexal torsion, but the AUC, sensitivity, and specificity results lead us to believe that studies with a larger sample size could help achieve the intended objective. Consequently, D-dimer levels could help surgeons in more accurately predicting the possibility of an adnexal torsion in children and adolescents, but this should not replace imaging studies and diagnostic laparoscopy especially in doubtful cases.

However, our study had several limitations. Firstly, the number of patients enrolled during the study period (*n* = 15) is small and limits statistical power. Therefore, the present study represents a pilot analysis waiting for results from the multicenter data and encouraging prospective randomized studies with larger sample sizes to verify our hypothesis. Moreover, time from onset of symptoms, hormonal fluctuations, nutritional and dietary factors, or eventual infections were not evaluated as possible confounding factors in this prospective pilot analysis.

Adnexal torsion currently remains a surgical emergency. We believe that it is crucial to identify pertinent clinical, laboratory, and imaging findings that enable surgeons to confidently establish an adnexal torsion diagnosis and select appropriate patients for surgical intervention, thereby avoiding unnecessary procedures, especially in pediatric patients. Our study highlights the potential diagnostic utility of preoperative serum D-dimer levels in diagnosing adnexal torsion, supporting their potential use in the early identification of this condition even if it cannot still replace imaging and laparoscopy to date.

## Data Availability

The raw data supporting the conclusions of this article will be made available by the authors, without undue reservation.
